# Forest fragmentation and impacts of intensive agriculture: Responses from different tree functional groups

**DOI:** 10.1371/journal.pone.0212725

**Published:** 2019-08-01

**Authors:** Juliana C. Tenius Ribeiro, André Felippe Nunes-Freitas, Elaine Cristina Cardoso Fidalgo, Mariella Camardelli Uzêda

**Affiliations:** 1 Departamento de Ciências Ambientais, Instituto de Florestas, Universidade Federal Rural do Rio de Janeiro, Seropédica, Rio de Janeiro, Brasil; 2 Centro Nacional de Pesquisa de Solos, Empresa Brasileira de Pesquisa Agropecuária, Rio de Janeiro, Rio de Janeiro, Brasil; 3 Centro Nacional de Pesquisa de Agrobiologia, Empresa Brasileira de Pesquisa Agropecuária, Seropédica, Rio de Janeiro, Brasil; University of Michigan, UNITED STATES

## Abstract

Agricultural landscapes are seen as areas of extreme importance for studying and developing strategies which integrate biodiversity conservation and ecosystem services with food production. The main strategies for intensifying agriculture are based on conventional agricultural practices of frequently using inputs for fertilization and correcting soil pH. Some studies show that these practices generate impacts on nearby forest fragments through soil contamination and increasing nutrient content. The objective of this study was to identify the impacts on the functional groups of sciophilous (late successional/shade-tolerant species) and heliophilous (pioneer/sun-loving) species of a tree community of 14 forest fragments near pasture areas and agricultural areas under conventional practices, raising the hypothesis that higher-fertility forest fragments adjacent to intensive agriculture modify the floristic composition of the tree community. Consequently, this study is based on the following questions: i) Do forest fragments within intensive farming environments present differences in floristic composition of species?; ii) Does the soil fertility influence the tree species composition?; iii) Which variables influence species abundance and richness in the forest fragments with different types of use around their environment? The floristic composition of fragments close to agricultural areas are more similar to each other than the composition of fragments close to pasture areas. Furthermore, the General Linear Model (GLM) results show a clear influence of the intensive farming environment on the richness and abundance of the two functional groups in the forest fragments, directly benefiting the abundance of heliophilous species, which are also benefited by the greater declivity and smaller fragment area, while the abundance of sciophytes is negatively correlated with these last two variables. The increase of calcium content is beneficial for the richness of heliophilous species, while the increase in phosphorus content influences a reduction in the richness of sciophyte species, which also strongly respond to the isolation between fragments. The results indicate a dominance trend of pioneer species in fragments with nutritionally enriched soils, providing evidence that the intense adoption of inputs in cultivated areas causes concrete impacts on the diversity of the tree community.

## Introduction

With the global demand for increased food production, agriculture is in increasing world expansion [1,2], mainly in tropical countries [3]. This is one of the main activities causing reduction and fragmentation of natural forests worldwide [4] and occupies almost 40% of the soil throughout the planet [5]. Thus, agricultural landscapes are today seen as areas of extreme importance for studying and developing strategies that integrate biodiversity conservation and ecosystem services with food production [2,6]. The main concern with this reality is focused on tropical regions which have the world’s highest biodiversity and the greatest pressure for increasing agricultural land [7,8]. It raises the need to develop production models that not only take into account greater production efficiency, but also the externalities that are implied on the biodiversity and ecosystem services of a landscape [2,6,9–12].

One of the main consequences of fragmentation is the edge effect [13–15], which is generated by creating borders between forest and agricultural ecosystems. The creation of this abrupt border is the main cause of alterations in the microclimate, the soil fertility and the presence of chemical substances from the surroundings in the borders and interior of the fragments [15,16]. This has short- and long-term effects on species composition [13,17–20], often favoring the proliferation of generalist and pioneer species [21–23] and hardy exotic species [22–25].

Edge effects are generally more intense in fragments of smaller area and with greater edge ratio [14,22,26]. However, the intensity and types of edge effects have direct connection with the characteristics and usage intensity in the environment [15,23,27].

The main practices for agricultural intensification are fertilization and pH correction which demands a frequent use of chemical inputs [1,2]. Some studies show that the intensification of these practices in agricultural fields causes changes in soil chemical characteristics on nearby forest fragments through contamination by fertilizers. [28,29]. This contamination occurs via fertilized soil particles in the crops being transported by wind to the fragments. These changes in fertility levels of soils in forest fragments can have significant impacts on the floristic composition due to the great influence of soil chemical characteristics on vegetation composition and spatial distributions [30–36].

Some nutrients are limiting to the growth of trees in forest environments [37–40], and the continuous increase of fertility levels in forest fragment soils may lead to alterations in soil chemical relationships [39–41], and possible species losses due competition and mortality [42,43]. Some studies suggest that some species such as pioneers are more efficient in using nutrient surpluses, with an increase in growth rates [33,39,40,43]. Thus, changes in soil fertility conditions along with other impacts associated with forest fragmentation may favor the establishment of pioneer species to the detriment of late successional species.

This study was conducted in a region of Atlantic Forest which, like most tropical forests, has developed on naturally acidic and nutrient poor soils. The vegetation of these forests developed with high species diversity and adapted to these chemical characteristics of the soil through mechanisms which enabled better absorption and utilization of nutrients and tolerance to high levels of aluminum [36].

A recent study showed increasing natural vegetation in the Atlantic Forest in recent years, however this biome still remains as a biodiversity hotspot [44]. This is because the native vegetation remains extremely fragmented amidst a modified matrix such as agriculture and livestock-producing landscapes, and much of the vegetation is low quality with a predominance of degraded secondary fragments [44,45].

Thus, the present study aimed to identify the impacts on the functional groups of sciophilous and heliophilous species in the tree community of forest fragments in the Atlantic Forest near agriculture areas with conventional practices of intensive fertilizer use. Pioneer or heliophilous species are those which require direct light for seed germination, or those generally classified as pioneers and early secondary. These species are the first colonizers of an environment in early ecological succession stages or which are common in altered natural environments. The non-pioneer/late successional or sciophilous species are those which can germinate and develop under shade, being found under the canopy [46]. It was hypothesized that the higher fertility of forest fragment soils adjacent to intensive agriculture modifies the floristic composition of the tree community, adopting the premise that there are nutrients added into the soils of these fragments [29].

Studies which aim to observe vegetation patterns with isolated factors such as the chemical characteristics of the soil present limitations due to the great correlation between the soil parameters and the vegetation itself, with it being greatly important to consider the landscape aspects, especially when it comes to heterogeneous landscapes [20]. These would include several factors related to forest fragmentation, water availability, and others [30,32,47]. Therefore, in order to identify the impact of a change in the fertility levels within forest fragments on the floristic composition of these sites, the vegetation correlation with local factors related to the soil also needs to be evaluated. These factors such as slope, soil grain size and canopy opening, as well as factors related to forest fragmentation such as size, isolation and shape of the fragments directly affect the floristic composition. Thus, this study was guided by the following questions:

- Do forest fragments within intensive farming environments present differences in floristic composition of species?
- Does the soil fertility influence the tree species composition?
- Which variables influence the species abundance and richness in the forest fragments with different types of use around their environment?

## Materials and methods

### Site description

The study was carried out in the Guapi-Macacu Basin in the state of Rio de Janeiro, located east of Guanabara Bay. The predominant climate in the region is Af tropical wet according to the Köppen classification. The average precipitation varies between 1,300 and 2,200 mm and the temperature between 14 and 27°C, presenting an average of 21.1°C [48]. The vegetation of the region is Dense Ombrophilous Lowland Forest included in the Atlantic Forest (sensu stricto) [49]. The forest cover of the basin occupies 42.4% of the territory and is divided into larger and continuous fragments in areas of higher elevation, while hills and hillocks are located in the lowlands with smaller and more dispersed forests in the landscape [48].

The study was developed in 14 forest fragments dispersed in the Basin, with an environment predominantly consisting of agriculture and livestock. The fragments were selected in four size ranges: 4 small fragments (< 15 ha), 4 medium fragments (> 19 < 30 ha), 3 large fragments (> 90 < 200 ha) and 3 segments of continuous forests (> 40,000 ha) belonging to the *Parque Estadual dos Três Picos* forest. Continuous forest segments were used to control fragmentation effects. The Atlantic Forest fragments selected for this study have been fragmented for at least 18 years, and their selection followed some criteria such as the native vegetation structure (given by stratification, tree size and life forms), management and usage history. There has been a great effort in identifying fragments of similar structure and with similar dynamics of use in their surroundings in the last ten years.

**Fig 1:**
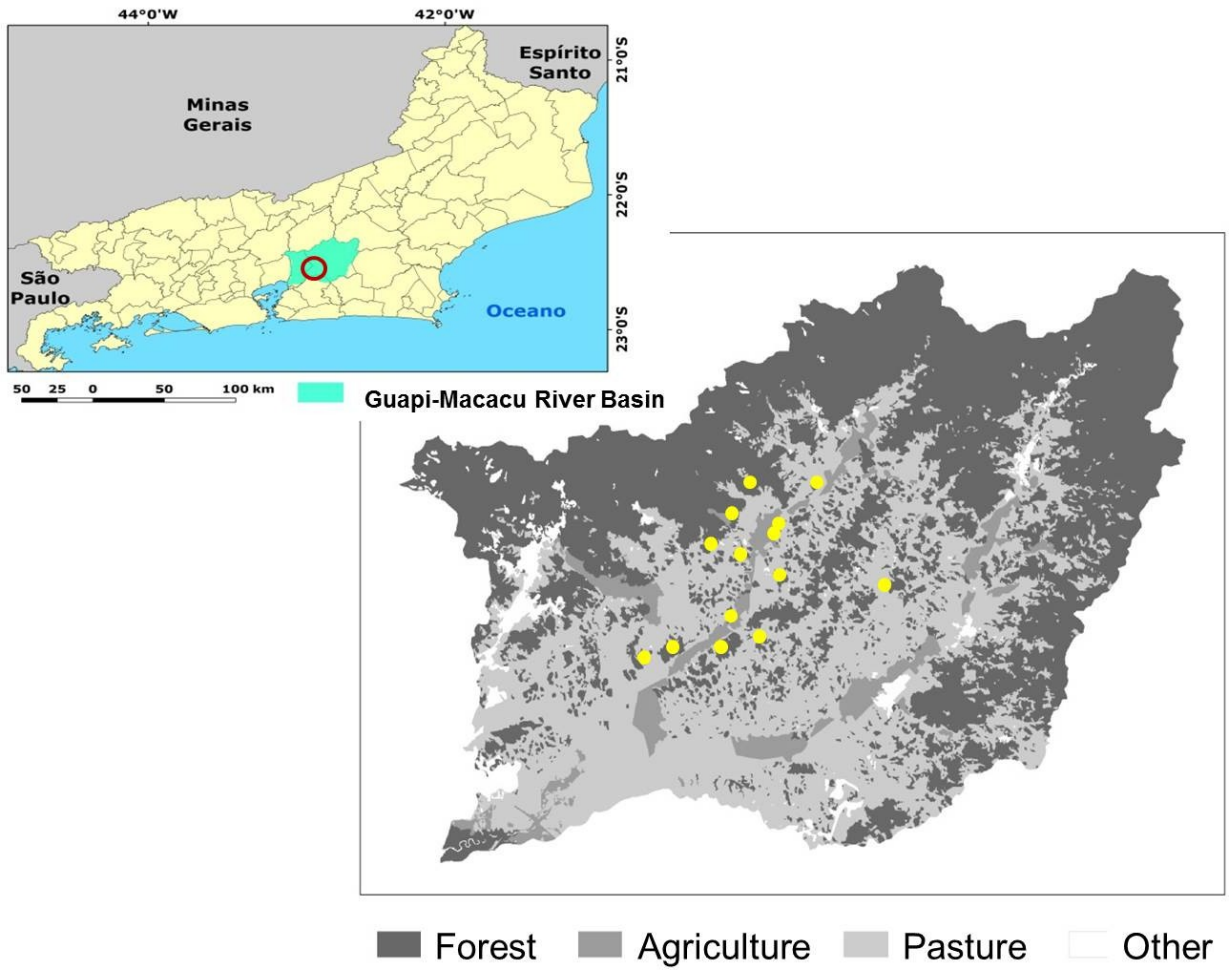
Location of the studied fragments and land use classes in the Guapi-Macacu River Basin, Rio de Janeiro, Brazil.

The fragments are surrounded by different soil uses, predominantly pastures and crops (43.6% and 4.8% of the territory, respectively) (Fig 1). Agriculture herein will be treated as intensive crop, with high use of agricultural inputs and soil tillage, and pastures.

All the forest fragments used in this research work were located in private properties. All the owners were contacted and gave permission for the researchers involved in this work to enter and to collect data prior to the field collections. No specific permits from local authorities were necessary because there was no need for tree cutting or intervention to any endangered or protected species.

Intensive use areas are characterized by annual corn (*Zea mays*) rotations with manioc cultivation (*Manihot esculenta*), with high frequency of soil rotation through plowing followed by harvesting and use of fertilizers and lime. An average of 2000 kg.ha^-1^ lime and 60 kg^-1^ of 4-14-8 NPK fertilizer are added for the corn cultivation. Some proprietors use organic compost (manure) applied as cover as a complement to chemical fertilization. Agrochemicals are also frequently used to control pests such as caterpillars (*Spodoptera frugiperda*). After this, new plowing and harrowing is carried out for planting the manioc roots, without new chemical fertilization. Management activities are almost non-existent in pasture areas (*Brachiaria brizantha*), with only general grazing occurring. The maximum stocking density of cattle is on average 1 head.ha^-1^.

The criteria described by Laurance et al. (2002) [50] were adopted as references to evaluate the effect of the soil use on the native vegetation areas. These authors establish that the anthropic area of the environment must have a minimum size of 100 m in length and the same area of width in contact with the fragment edge.

### Sampling design

Six fragments were evaluated for an adequate evaluation of the established questions, with an agricultural percentage in their perimeter being zero, and 5 fragments and 3 areas of continuous forest (part of the *Parque Estadual dos Três Picos*) with a variable percentage in their perimeter surrounded by agriculture covering all size classes of the selected fragments. All the matrices classified as intensive crops have a continuous usage history for at least 10 years.

The selected forest fragments are characterized by a sloping hillside relief, typically uphill from the edge of the forest toward the interior, and thus three different strata were established for sampling, with the sole objective of sampling the differences in slope and distances from the edge of the fragment to the production areas. The fragments were stratified as follows: i) 1^st^ third—area closer to the agroecosystem, which has greater anthropic influence; ii) 2^nd^ third–the area next to the 1^st^ third, generally with great slope; iii) Final third—top hill region, with lower slopes. Three plots of 50 x 5 m (250 m²) were allocated in each third, which totals an area of 2,250 m² per fragment. The plots were systematically delimited in each stratum, parallel to the agroecosystem, with 10 meters of distance between them in the horizontal direction (parallel to the edge) and 10 meters in the vertical direction (perpendicular to the edge and towards the inside). Three plots were also allocated in the surrounding agricultural areas for soil sampling, following the same methodology for allocating the plots within the fragments.

### Floristic and phytosociological survey

A forest inventory was carried out in March 2016 with assistance from a botanist. All individual trees with diameter at breast height (DBH) greater than or equal to 5 cm were measured and identified in all the plots. When possible, the identification was carried out in the field and, when necessary, botanical material collections were carried out for further identification. The taxonomic determinations were carried out by consulting the Herbariums of the Research Institute of the Botanical Garden of Rio de Janeiro and the Department of Botany (RBR) of UFRRJ (Federal Rural University of Rio de Janeiro), by bibliography, or by specialists. Part of the floristic survey had already been carried out in a previous study [29] using the same methodology. These data were also collected for conducting this work.

The species were classified into two functional groups according to Swaine and Whitmore [46]: pioneer or heliophilous species, which require direct light for seed germination, or those generally classified as pioneers and early secondary; and the group of non-pioneer/late successional or sciophilous species which can germinate and develop under the shade, being found under the canopy and also in open environments [51]. Studies for the state of Rio de Janeiro were used for these classifications in order to avoid variations in classifications [51–55]. Non-classified species (NC), did not fall into any of the categories due to lack of information.

The calculations of the community phytosociological parameters were performed according to Mueller-Dombois & Ellenberg [56] by calculating the absolute and relative parameters of species density, dominance and frequency in order to obtain the Importance Value (IV) of each species, which consists of the sum of the relative values mentioned above (S1 Table).

### Soil fertility and granulometry

Simple soil (different amounts of soil taken at different points within the plot areas) samples were collected between February and March 2016 in order to analyze the relationship between abundance and richness of functional groups of tree species and the soil fertility characteristics of the forest fragments. Three soil samples were collected per plot at a depth of 0–5 cm with the aid of a metal probe. Samples were collected at the ends (0; 50 m) and center (25 m) of each plot, forming a sample composed of soil for each plot. Each composite sample was placed in a plastic bag and identified for transport to the laboratory, where each soil sample was air dried. After drying, they were sieved using an 8 mm sieve to remove coarse material. The samples were sent for chemical analysis in the Nutrient Cycle Laboratory of EMBRAPA Agrobiology, which follows the methods recommended by EMBRAPA [57]. The chemical characteristics analyzed were pH in water, calcium (cmolc.dm^-3^), magnesium (cmolc.dm^-3^), potassium (mg.L^-1^), phosphorus (mg.L^-1^), carbon (dag.Kg^-1^) and nitrogen (dag.Kg^-1^). The granulometric analysis of the samples was carried out at the Soil Physics Laboratory of the *UFRRJ* Soils Department, using the Pipette Method [57], quantifying the sand, silt and clay fractions expressed as percentage.

### Canopy opening

Hemispheric photographs were taken for estimating the canopy cover of each fragment, from which it was possible to indirectly calculate the canopy cover and light input in the plots [58]. The photographs were taken at the ends (0; 50 m) and center (25 m) of each plot using a digital camera attached to a ‘fish eye’ lens positioned at a distance of 1.5 m from the ground using a tripod. The tripod was always positioned to the north with the help of a compass in order to maintain standardization of the photographs. Afterwards, each photo was analyzed with the purpose to quantify white (the points related to the open sky) and black (referring to the vegetation) pixels, which was performed using the Gap Light Analyzer (GLA), Version 2.0 program [59]. Analysis of the photographs enabled an estimation for each plot for the canopy opening, direct light input and diffuse light. Direct light input was used as a measure of canopy opening for the statistical analysis of the present study due to the high correlation of these parameters.

### Slope

The central point in each plot (25 m) was measured with the aid of a digital clinometer for evaluating the terrain slope.

### Obtaining and evaluating landscape indexes

In order to understand the relationships between the structural variables of the landscape and the tree community composition in the fragments, landscape metrics were used according to the procedures used in Uzêda et al. [29]. The following measures were used: area, which refers to the fragment size; perimeter/area ratio (PARA), which is an indicator of the fragment shape, and therefore related to the amount of existing border; and Euclidean distance of the nearest neighbor (ENN), which is an indicator of fragment isolation. The agricultural border percentage (agri) was also obtained, which is an indicator of the border perimeter percentage of the forest fragment which is directly in contact with the agricultural area. All types of land use along the fragment perimeter that made direct contact with its border which were delimited in the map and coverage used were considered for this calculation.

The land use and coverage map of the Guapi-Macacu and Caceribu river basins on a scale of 1:50.000 [60] was used to identify the areas under natural vegetation cover in the study area. This map was elaborated based on the image classification of the TM-Landsat 5 sensor, from June to August 2007, with a resolution of 30 meters. The original map was recorded to the boundary of the Guapi-Macacu basin area, and the selected natural vegetation sites were extracted: forest in initial, middle and advanced stages of regeneration. The fragment data were spatialized in digital format (“raster”) with a resolution of 30 meters, and the Fragstats program was used [61] for calculating landscape metrics.

Next, the proportion of the fragment boundaries to be found in line with the different types of land use was calculated. As the studied fragments are only surrounded by pasture and agriculture, and therefore the percentages of these two edge types total 100, it was decided to only use the border percentage with agriculture, thus avoiding highly correlated variables [29].

### Data analysis

In order to identify the similarity of the structure and composition of the tree community among the fragments, non-metric multidimensional scaling (NMDS) with the Bray-Curtis similarity index [62] was used to verify clustering trends. Therefore, a matrix with the Importance Value (IV) of the species of each fragment (S2 Table) was used. NMDS construction was performed in the statistical R program using the “vegan” packages, version 2.3.0, and “labdsv” version 1.7.0 [63].

Generalized linear models (GLMs) were constructed and tested to identify the variables that best explain the functional groups’ abundance and richness pattern in the fragments. Therefore, in order to construct these predictive models, the richness and abundance of the ecological groups (pioneer and shade-tolerant) were considered as dependent variables and the levels of carbon, nitrogen, phosphorus, potassium, calcium and magnesium (C, N, K, Ca and Mg, respectively), clay percentage in the soil (Arg), border percentage with agriculture (lagri), Euclidean distance of the nearest neighbor (ENN), fragment size in hectares (area), perimeter-area ratio (PARA), canopy opening (abos) and slope as independent variables (S3 and S4 Tables). We used abundance of each species in each ecological group and average data of the independent variables in each stratum of each fragment as inputs. Next, 19 possible models were tested for the abundance of heliophytes and 28 models for the abundance of sciophytes in total. Then, 33 models were tested for the richness of heliophilous species, and 27 models for the richness of the sciophilous species (S5 Table).

Using Gaussian data distribution, the models were tested and selected through the second order Akaike criteria (AICc) generating a rank from the best to the worst model [64]. Models with ΔAICc values smaller than two (ΔAICc < 2) and of higher AICc weight (AICcWi) were selected. AICcWi is the selection probability of a given model in the cases of re-sampling the available data. Verifying the likelihood of the parameters to the selected models was performed by a chi-squared test (χ²). The analyses of the models were carried out in the R statistical software program [63] using the packages “bbmle”, version 1.0.16, and “MuMIn”, version 1.15.1.

## Results

A total of 4926 individuals were sampled from trees and palms, and 371 morphospecies were identified in 58 botanical families for the 14 forest fragments (total sampling area of 3.15 ha) studied in the Guapi-Macacu Basin. Of the morphospecies, 242 (66.58% of the total) were identified at the specific level, 61 (16.44%) at the genus level, 36 (9.70%) at the family level and 27 (7.28%) remained undetermined (S1 Table). The richness of the fragments ranged from 50 species to 109 species.

**Fig 2:**
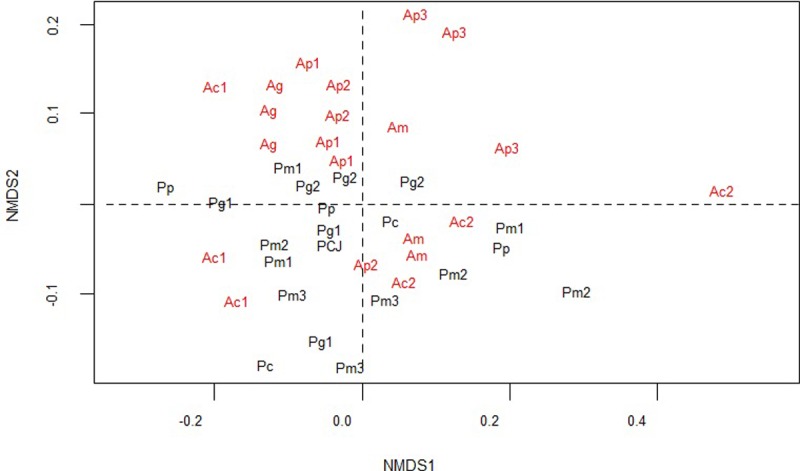
**Multidimensional non-metric scaling of the structure and composition of the tree species community in fragments of small (p), medium (m), large (g) and continuous (c) sizes, adjacent to intensive agricultural environments (A) and pastures (P). Fragments neighboring agricultural fields (IC) are in red and fragments neighboring cattle grassland (EC) are in black**. *Stress* = 0.11; R^2^ = 0.966.

The non-metric multidimensional scaling (NMDS) of the tree species community composition of the fragments (Fig 2) reflected the tendency of the sites belonging to fragments adjacent to the pastures areas to be grouped, predominantly in the lower quadrants of the graph, whereas the fragments near intensive agriculture are concentrated in the upper quadrants.

Regarding the abundance of the heliophilous species, GLM indicated that the greater extent of direct edge with agriculture (p < 0.001) and higher slope (p < 0.01) positively affected the number of individuals, while the higher magnesium levels (p < 0.05) and the increase in the size of the fragments (p < 0.05) had a negative relation with the number of individuals in this functional group. Three explanatory models (Table 1) were selected for the abundance of heliophiles, in which the variables of agriculture border percentage, fragment size, slope and calcium and magnesium contents were included, and the latter two factors were not significant by the chi-square test. The predictive model with the highest weight (AICcWi = 0.3167) showed the agriculture border percentage (p < 0.001) and the slope (p < 0.01) negatively interact in the abundance and the fragment size (p < 0.1) has a negative effect on heliophile abundance. The second model (AICcWi = 0.1686) also presented the magnesium content (p < 0.05) as explanatory, negatively interacting in the number of tree individuals in the heliophilous group.

**Table 1:** Results of the selected GLM models (ΔAICc < 2) to explain the abundance of heliophilous species in the forest fragments of the Guapi-Macacu Basin. The independent variables, the term used in the model, their respective coefficients (95% CI) and their significance by the Chi-square test (χ^2^) are specified below the models.

Model/Parameters	Term	Coefficient	χ²
Heliophilous = β0 + β1 agri + β2 area + β3 slope		ΔAICc = 0	AICcWi = 0.3167
Intercept	Intercept	145.900	
Agri border percentage	agri	576.40	0.0001708 ***
Fragment size	area	-0.41	**0.0511857 #**
Slope	slope	1.478	0.0052936 **
Heliophilous = β0 + β1 Ca + β2 Mg + β3 agri + β4 area + β5 slope		ΔAICc = 1.1	AICcWi = 0.2113
Intercept	Intercept	165.000	
Calcium	Ca	56.410	0.110645
Magnesium	Mg	-138100	0.046768 *
Agri border percentage	agri	597.90	6.792e-05 ***
Fragment size	area	-0.56	0.010340 *
Slope	slope	1.623	0.003206 **
Heliophilous = β0 + β1 Mg + β2 agri + β3 area + β4 slope		ΔAICc = 1.3	AICcWi = 0.1686
Intercept	Intercept	15.4200	
Magnesium	Mg	-60910	0.22489
Agri border percentage	agri	605.70	8.551e-05 ***
Fragment size	area	-0.49	0.025151 *
Slope	slope	1721	0.002357 **

*** Significant at 0.1%,

** 1%,

* 5% and #10% probability.

Next, four explanatory models were selected for the abundance of the sciophilous species (Table 2). Only the increase in the area of the fragments presented a positive relation with the abundance of these shade tolerant species (p < 0.05), while the slope was the only factor that presented a negative relation with the abundance of individuals in the sciophilous group (p < 0.05). The agricultural border percentage, phosphorus and calcium content variables were not significant by the chi-square test in these models.

**Table 2:** Results of the selected GLM models (ΔAICc < 2) to explain the abundance of sciophilous species in the forest fragments of the Guapi-Macacu Basin. The independent variables, the term used in the model, their respective coefficients (95% CI) and their significance by the Chi-square test (χ^2^) are specified below the models.

Model/Parameters	Term	Coefficient	χ²
Sciophilous = β0 + β1 P + β2 area + β3 slope		ΔAICc = 0	AICcWi = 0.2146
Intercept	Intercept	7.97E+08	
Phosphorous content	P	-4.46E+07	0.12067
Fragment size	area	3.39E-04	0.04164 *
Slope	slope	-0.78	**0.05859 #**
Sciophilous = β0 + β1 agri + β2 area + β3 slope		ΔAICc = 0.6	AICcWi = 0.1616
Intercept	Intercept	6.30E+08	
Agri border percentage	agri	-0.15	0.17479
Fragment size	area	0.00	0.02455 *
Slope	slope	-0.89	0.02994 *
Sciophilous = β0 + β1 Ca + β2 area + β3 slope		ΔAICc = 1.8	AICcWi = 0.0857
Intercept	Intercept	6.08E+04	
Calcium content	Ca	-1.56E+04	0.44894
Fragment size	area	0.3878	0.02050 *
Slope	slope	-788.1	**0.07184.**
Sciophilous = β0 + β1 P + β2 agri + β3 area + β4 slope		ΔAICc = 2.0	AICcWi = 0.0792
Intercept	Intercept	7.71E+08	
Phosphorous content	P	-3.48E+07	0.25275
Agri border percentage	agri	-1.01E-01	0.38941
Fragment size	area	3.34E-04	0.04283 *
Slope	slope	-7.94E-01	**0.05170 #**

*** Significant at 0.1%,

** 1%,

* 5% and #10% probability.

Two predictive models were selected for the richness of the heliophilous species (Table 3). Both the higher weight model (AICcWi = 0.3066) and the lower weight model (AICcWi = 0.1768) indicated calcium (p < 0.01) and soil clay levels (p < 0.001) as positive, and the greater opening of the canopy (p < 0.001) as negative.

**Table 3:** Results of the selected GLM models (ΔAICc < 2) to explain the richness of heliophilous species in the forest fragments of the Guapi-Macacu Basin. The independent variables, the term used in the model, their respective coefficients (95% CI) and their significance by the Chi-square test (χ^2^) are specified below the models.

Parameters	Term	Coefficient	χ²
Heliophilous = β0 + β1 Ca + β2 Cano + β3 Clay		ΔAICc = 0	AICcWi = 0.3066
Intercept	Intercept	15.52	
Calcium content	Ca	9.04	0.0011481 **
Canopy opening	Cano	-0.2094	0.0002689 ***
Clay content	Clay	0.2049	0.0005280 ***
Heliophilous = β0 + β1 Ca + β2 Clay + β3 ENN + β4 Cano		ΔAICc = 1.1	AICcWi = 0.1768
Intercept	Intercept	14.95	
Calcium content	Ca	8.59	0.0016990 **
Clay content	Clay	0.20323	0.0004671 ***
Isolation	ENN	0.01462	0.2013926
Canopy opening	Cano	-0.24649	0.0001455 ***

*** Significant at 0.1%,

** 1%,

* 5% and **#** 10% probability.

Four models were selected for the richness of the sciophilous species (Table 4), wherein only the magnesium content showed a positive interaction (p < 0.1), while the higher isolation factors of the fragment (p < 0.01), the increase in phosphorus levels (p < 0.1) and the higher slope (p < 0.1) were negative. The highest weight model (AICcWi = 0.1614) only presented the isolation variable of the fragments (p < 0.05) as explanatory for the species richness of the sciophilous group. In addition to the negative interaction of isolation (p <0.01), the second model (ΔAICc = 0.1; AICcWi = 0.1522) showed negative interaction with the increase in phosphorus content (p < 0.1) and positive interaction with magnesium content (p < 0.1). The third model (AICcWi = 0.1450) added the terrain slope (p < 0.1) as a negative explanation. Finally, the fourth model (AICcWi = 0.1241) only presented isolation (p < 0.01) as a negative factor for sciophyte richness, just as the first model.

**Table 4:** Results of the selected GLM models (ΔAICc < 2) to explain the richness of sciophilous species in the forest fragments of the Guapi-Macacu Basin. The independent variables, the term used in the model, their respective coefficients (95% CI) and their significance by the Chi-square test (χ^2^) are specified below the models.

Model/Parameters	Term	Coefficient	χ²
Sciophilous = β0 + β1 ENN		ΔAICc = 0	AICcWi = 0.1614
Intercept	Intercept	13.81	
Isolation	ENN	-0.04222	0.01327 *
Sciophilous = β0 + β1 P + β2 Mg + β3 ENN		ΔAICc = 0.1	AICcWi = 0.1522
Intercept	Intercept	15.42	
Phosphorous content	P	-60910	**0.063508 #**
Magnesium content	Mg	605.70	**0.083546 #**
Isolation	ENN	-0.49	0.004779 **
Sciophilous = β0 + β1 Mg + β2 ENN + β3 slope		ΔAICc = 0.2	AICcWi = 0.1450
Intercept	Intercept	14.4	
Magnesium content	Mg	13.73	**0.07294 #**
Isolation	ENN	-0.04262	0.00850 **
Slope	Slope	-0.15919	**0.06733 #**
Sciophilous = β0 + β1 P + β2 ENN		ΔAICc = 0.5	AICcWi = 0.1241
Intercept	Intercept	18.06	
Phosphorous content	P	-0.814630	0.165476
Isolation	ENN	-0.04613	0.006773 **

*** Significant at 0.1%,

** 1%,

* 5% and

**#** 10% probability.

## Discussion

### Impacts of intensive agriculture on functional groups

The predictive model results point to a clear and direct influence of the intensive farming environment on the tree community in the forest fragments. This can be observed by the influence that the greater direct border percentage with agriculture areas has on the abundance of the functional groups. According to the models, the forest fragments with greater border extension focused on intensive corn and manioc cultivation are associated with greater pioneer species abundance and the lowest abundance of sciophilous species. Furthermore, there seems to be possible indirect impacts caused by the soil eutrophication inside the fragments, possibly due to the aerial drift of fine particles of the soil which are eventually deposited inside these fragments. Thus, the direct edge of a forest fragment with an intensive agriculture area has potential impact on the abundance and richness of the functional groups due to differences in interaction between functional groups and fertility levels. As indicated by the models, the heliophytes are benefited by calcium levels and disadvantaged by magnesium levels, while the sciophilous species are benefited by magnesium and are disadvantaged by the higher phosphorus levels.

Among the potential impacts from surrounding agricultural areas on the interior of the fragments, changes in soil fertility with increased nutrient levels have been widely discussed in the literature [23,28,29,65]. A recent work [29] showed a trend of soil eutrophication in forest fragments located in the same study area, with significant increases in calcium, phosphorus and potassium, while another similar study [65] observed that the higher the fertilizer application intensity in agricultural areas, the higher the nutrient content in nearby forest fragments. The frequent practices of liming and fertilizer use in conventional agriculture fields combined with soil tilling result in drift and transport of fine soil particles by air, which are deposited in soil fragments [28,29,65]. The result is a significant increase in nutrient levels in the soils of nearby forest fragments such as nitrogen [30,65], phosphorus [28,29], calcium and magnesium [23,29].

The relationship between the abundance and richness of early and late successional species and the increase in calcium, magnesium and phosphorus nutrient contents found in this study demonstrate that the enrichment of nutrients in the soils of these fragments by the practices implemented in their surroundings is a source of important impact in the tree community and which is still little recognized. This was observed in some studies that identified changes in the floristic composition of herbaceous leaves at the edges of fragments, possibly due to the large input of liming particles for soil acidity correction and the use of nitrogenous fertilizers, causing a dominance of common species in less acidic soils or of nitrophilous species [23,66–69].

Pioneer tree richness is clearly benefited by increased calcium content, while late successional species are negatively impacted by increased phosphorus content. These nutrients tend to increase in forest fragments near agriculture areas [23,29,65] due to the common practice of liming and use of phosphate fertilizers. In addition, phosphorus has low mobility in the soil, which can cause accumulation, and consequently lead to eutrophication of the system over time [29]. Although magnesium content has a positive relationship with the richness of sciophytes, the previous results found in this same study area [29] did not indicate significant increases of this nutrient in the fragments, which is the opposite of that found for calcium and phosphorus, as previously mentioned.

Thus, continuous additions of nutrients over long periods may have significant impacts on species composition of the forest community of forest fragments [37] close to conventional agricultural crops, altering the composition and structure of the functional groups of the tree community. Natural ecosystem plants are adapted to conditions with limited availability of nutrients, maintaining a certain balance of adjustment to local fertility characteristics [36,37]. Thus, soil fertility conditions in tropical forests limit plant growth and establishment, therefore constituting an important determinant of functional diversity on a local, landscape and regional scale [30,32,37].

Some recent long-term studies have investigated the primary productivity response of tropical rainforest trees to adding nutrients to the soil [37–41], demonstrating that even in soils relatively rich in exchangeable bases (K^+^, Ca^2+^, Mg^2+^ and Na^+^) and in nitrogen and phosphorus, some species and functional groups respond strongly to the addition of these elements and potassium. However, the impact in regions with lower fertility such as in the areas of this study may be more intense, since these regions tend to have more specialized flora when compared to regions with higher fertility, which have a greater number of fast-growing and more generalist tree species [32,37]. The literature demonstrates that fast-growing species are generally more abundant in naturally nutrient-poor soils [33,43]. This is because the availability of nutrients is not a limiting factor for pioneering and fast growing plants as their wood is low density, which does not require a high investment in nutrients [35,43]. Therefore, pioneer species seedlings are more efficient in nutrient consumption, being able to out-compete shade tolerant species in competitive environments [70].

Thus, constant deposition of nutrients in the forest fragment soils of this study may be creating a conducive environment for the proliferation of pioneer tree species. The fragments within the pasture environment showed the highest IV values for the sciophilous species (S1 Table), which helps to explain the observed grouping of these fragments in the ordering analysis. Thus, it is possible to observe that the pioneer or secondary heliophilous species may be benefiting in the fragments within an intensive agriculture environment, where the effects of nutrient drift are greater. When analyzing the two functional groups (heliophilous and sciophilous), it is possible to observe that fast growing pioneer species generally present higher rates of primary production in response to nutrient increase [4,70,71].

However, despite the influence of changes in soil fertility demonstrated in the results of our study, this variable does not solely explain the composition of the functional diversity in the fragments. The environment can affect the composition of the species in two ways: firstly by edge effects, which are more intense when the environmental matrix is agricultural, changing the chemical characteristics of the soil and creating direct impacts on the microclimate; and second, by factors that hinder the recruitment of late successional species by reducing propagule flow [4,14,72–74].

Agriculture areas may also impact shade tolerant species by influencing the connectivity between the fragments, as they may be a more hostile environment for the movement of pollinators and dispersers [14,75], reducing seed flows [74]. Changes in the number of individuals of some species due to decreased seed dispersal can cause local extinctions over time [74], making it difficult to maintain viable populations. In addition, more intensive use in agricultural areas may create unfavorable microclimatic conditions to the floristic community [76], and may contribute to the impact on the abundance of some shade tolerant species.

### Other impacts on functional groups

The higher abundance of sciophilous species was dependent on the fragment size, being the only factor that positively influences the abundance of this group. On the other hand, the abundance of pioneer species is greater in smaller fragments, which is directly related to the fact that these smaller fragments are subject to greater intensity of edge effects [22,77]. The fragment size is not solely the main factor for tree species abundance. Its indirect effect is related to other associated factors such as edge effects, the characteristics of the landscape matrix and the isolation between the fragments [8,77,78].

The impact of abrupt edge creation by the fragmentation process for tree communities has been widely discussed, resulting in the elevation in mortality rates and changes in the recruitment dynamics of later successional trees at the edges of fragments [17,76,79], and even in the interior of small fragments [26], benefiting pioneer and generalist species [17,19].

Despite the significant effect on abundance, the species richness for the two functional groups was not affected by the fragment size. For richness, the soil characteristics (fertility levels and clay content), the canopy opening, the slope and isolation degree of the fragments are more important. Plant diversity is highly related to soil fertility and grain size characteristics [30,32,80], and these two factors are strongly correlated as previously discussed. A higher clay content in the soil allows more bonds, which provides greater nutrient availability [31,32]. Along these lines, flatter lands have a higher amount of clay coming from higher lands and deposited in these lower areas [31,32], which in turn also results in higher levels of soil fertility.

In addition to the influence on soil fertility, the slope of the terrain may make it difficult to maintain sciophilous species in two ways; firstly, because the recruitment of these species is difficult as they usually have large seeds which may present greater difficulties in establishment in very sloped areas; secondly, greater slope is associated with tree falling and mortality of large trees, since they present greater difficulty to remain fixed in these areas [32,43]. These situations explain the difficulty in maintaining the seedling recruitment of slow growing species, showing the positive influence of this factor on the abundance of pioneer species, and a negative relation with the abundance and also richness of sciophilous species.

The higher degree of isolation of the fragments did not influence the abundance of the species of the two ecological groups, but was one of the explanatory variables for the richness of late successional species, being more important than the fragment size [8,77]. As already discussed, this factor leads to reductions in seed flow, decreasing the chances of seed dispersal due to longer distances between fragments [4,72]. Initial successional species have an advantage in seed dispersion because they produce a greater quantity of seeds and generally their seeds are dispersed by wind or generalist animals [51]. On the other hand, late successional species generally produce larger seeds dispersed by specialist animals [15], making it harder for gene flows between more isolated fragments.

Finally, unlike what is expected, the larger canopy opening only had a negative influence on the richness of heliophilous species. This result differs from that expected, since greater abundance and diversity of light-loving species are expected for germination and growth in great clearings [34,81]. However, areas with larger canopy openings may have a lower species richness (even of heliophiles) due to different disturbances, regardless of soil fertility. This is also related to factors of the natural dynamics of succession in gaps, which may be totally different in small and large gaps. Even if there is a small opening created in the canopy from the falls of some trees or branches, regeneration will not necessarily be occupied by pioneer species [34].

The study sought to show the direct and indirect impacts of agriculture on the functional diversity of trees in forest fragments of the Atlantic Forest. In addition to the well-known edge effects, we tried to show that agricultural practices have an effect that has not yet been studied, such as changes in soil fertility of natural forest ecosystems and their implications on biodiversity. The use of an expressive number of forest fragments for comparing the intensive agriculture and pastures in the surroundings demonstrated that the conventional intensive agriculture can imply in a regression in the forest succession, either directly by the edge effects and alteration in soil fertility, and/or indirectly by the possible impact on the flows of dispersing and pollinating animals. However, as already shown, multiple factors negatively affect forest succession. In addition to the matrix effect, the fragmentation effects (size, isolation) and local variables (slope, canopy, clay content) cannot be ignored in this process [35].

Thus, forest fragments immersed in an agricultural matrix and exposed to conventional and intensive agriculture practices may be conditioned to a reduction in tree species diversity through the replacement of late successional species by pioneer species. Late successional species are responsible for most of the natural regeneration and the high diversity in mature tropical forests [19,36], and show a tendency to be restricted to areas with no edge effects reaching them, such as in interiors of large fragments and fragments with little isolation. Thus, the discussion of conservation in agricultural production landscapes should take into account that it is necessary to develop and encourage agricultural practices which are less impactful on biodiversity, in addition to the demand for maintaining large areas of natural remnants which is essential for some species. This is important to reduce the direct impacts caused by edge effects, such as the change in the fertility characteristics of the soils, and the impacts that the matrix quality can imply on the population flows between the fragments [22].

## Conclusion

This study indicates that the intense adoption of chemical inputs for fertilization in agricultural areas causes concrete impacts on the tree functional groups of forest fragments. This study shows that increasing soil fertility of forest fragments adjacent to intensive agriculture areas causes impacts on both abundance and functional groups richness of the tree community. The increase in phosphorus levels may cause a decrease in the richness of shade tolerant species, while the pioneers benefit from increased calcium levels and the impacts inherent to forest fragmentation such as size reduction and fragment isolation. In this way, a continuous increase in the populations of pioneer species may be causing a regression in the successional stage of these remnants. The ecological intensification of agriculture is a challenge and an emergency for fragmented agricultural landscapes, where the need for biodiversity conservation policies and ecosystem services must be linked to agricultural production. This underscores the demand for policies which support developing more conservation-oriented agricultural production strategies, and indicates potential negative externalities of adopting strategies based on the conventional and intensive agricultural system in the tropics, which are generally not considered.

## Supporting information

S1 Table Tree species sampled per strata in forest fragment and their respective basal area values and ecological group classification. (XLSX)

S2 Table Importance value (IV) matrix of each tree species at each sampling site. (XLSX)

S3 Table Abiotic factors values sampled at each site. C: carbon; N: nitrogen; P: phosphorus; K: potassium; Ca: calcium; Mg: magnesium; agri: border percentage with agriculture; area: fragment size in hectares; PARA: perimeter-area ratio; ENN: Euclidean distance of the nearest neighbor; Abos: canopy opening; Arg: clay percentage in the soil; decli: slope.
(XLSX)

S4 Table Landscape metrics at each fragment site. agri: border percentage with agriculture; area: fragment size in hectares; PARA: perimeter-area ratio; ENN: Euclidean distance of the nearest neighbor;
(XLSX)

S5 Table Tested models for prediction of richness and abundance of sciophilous and heliophilous tree species. (XLSX)
